# Branched Poly(*ε*-caprolactone)-Based Copolyesters of Different Architectures and Their Use in the Preparation of Anticancer Drug-Loaded Nanoparticles

**DOI:** 10.3390/ijms232315393

**Published:** 2022-12-06

**Authors:** Evi Christodoulou, Maria Notopoulou, Eirini Nakiou, Margaritis Kostoglou, Panagiotis Barmpalexis, Dimitrios N. Bikiaris

**Affiliations:** 1 Laboratory of Polymer Chemistry and Technology, Department of Chemistry, Aristotle University of Thessaloniki, 54124 Thessaloniki, Greece; 2 Laboratory of General and Inorganic Chemical Technology, Department of Chemistry, Aristotle University of Thessaloniki, 54124 Thessaloniki, Greece; 3 Department of Pharmaceutical Technology, School of Pharmacy, Aristotle University of Thessaloniki, 54124 Thessaloniki, Greece

**Keywords:** poly(ε-caprolactone), ring-opening polymerization, branched copolymers, nanoparticles, anticancer, paclitaxel

## Abstract

Limitations associated with the use of linear biodegradable polyesters in the preparation of anticancer nano-based drug delivery systems (nanoDDS) have turned scientific attention to the utilization of branched-chain (co-)polymers. In this context, the present study evaluates the use of novel branched poly(ε-caprolactone) (PCL)-based copolymers of different architectures for the preparation of anticancer nanoparticle (NP)-based formulations, using paclitaxel (PTX) as a model drug. Specifically, three PCL-polyol branched polyesters, namely, a three-arm copolymer based on glycerol (PCL-GLY), a four-arm copolymer based on pentaerythritol (PCL-PE), and a five-arm copolymer based on xylitol (PCL-XYL), were synthesized via ring-opening polymerization and characterized by proton nuclear magnetic resonance (^1^H-NMR), gel permeation chromatography (GPC), intrinsic viscosity, differential scanning calorimetry (DSC), X-ray diffraction (XRD), and Fourier-transform infrared (FT-IR) spectroscopy and cytotoxicity. Then, PTX-loaded NPs were prepared by an oil-in-water emulsion. The size of the obtained NPs varied from 200 to 300 nm, while the drug was dispersed in crystalline form in all formulations. High encapsulation efficiency and high yields were obtained in all cases, while FTIR analysis showed no molecular drug polymer. Finally, in vitro drug release studies showed that the studied nanocarriers significantly enhanced the dissolution rate and extent of the drug.

## 1. Introduction

Nanomedicine is an emerging discipline in pharmaceutical technology that focuses, amongst others, in the development of novel therapeutic approaches and innovative drug delivery formulations [1,2]. In this sense, various materials have been investigated in order to fabricate efficient and effective nanoparticle (NP) drug delivery systems (nanoDDS), including inorganic-based, viral-based, lipid-based, and polymer-based compounds [3,4,5,6,7,8,9,10,11]. Amongst them, polymeric materials (either of natural or synthetic origin) have gained much attention due to their high versatility, cost-effective production (mostly in the case of synthetic polymers or copolymers), and easily tunable properties [12,13,14].

Focusing more on anticancer nanotherapy requirements, the polymeric materials applied in the preparation of such nanoDDS must possess several unique characteristics, such as low toxicity (since most anticancer active pharmaceutical ingredients (APIs) are already highly potent compounds), controllable drug delivery, and a good biodegradation profile (i.e., being easily eliminated by the body) [3]. Consequently, the prepared nanoDDS must successfully encapsulate anticancer medicines (which, in most cases, are administrated in extremely low doses), be easily converted into a stable NP colloidal dispersion within the body, and simultaneously be stable during storage. In this context, although linear biodegradable polymers, such as polyacrylic acid (PAA), poly(lactic acid) (PLA), and poly(lactide-glycolide) (PLGA), have been extensively employed in the last few decades, they exhibit several limitations associated with their high polydispersity, low solubility, and coil-like morphology [15,16,17,18,19]. These limitations, which lead to low surface functionality and low encapsulation efficiency, have restricted their use in such nanoDDSs, turning, consequently, the focus of several pharmaceutical formulations and material scientists into the synthesis of new polymeric compounds [20,21,22,23].

In this vein, an appealing approach to overcome the aforementioned limitations of linear polymeric materials is to use branched (or hyperbranched) analogs [15]. In general, branched polymers (or copolymers) represent an attractive alternative to other similarly branched structures, such as branched polymeric micelles, comb-like compounds, and dendrimers [24,25,26,27,28,29,30]. Especially in the case of dendrimers (which provide a vast surface with functional groups that can be utilized as active sites to bind target molecules via chemical bonding, enabling them to efficiently deliver a wide variety of drugs), the use of branched polymers as alternatives can overcome the laborious multi-step synthesis and, consequently, the high manufacturing cost, while maintaining their significant advantages (such as three-dimensional globular structure, high polymeric cavities, high solubility, and high surface functionality) [15,31]. Moreover, when these new branched polymeric materials are based on polyesters, several additional advantages are also added, such as high aqueous stability, excellent biocompatibility, and easily tunable hydration and biodegradation characteristics [15].

Poly(e-caprolactone), PCL, is a hydrophobic, non-toxic, biodegradable (and, thus, biocompatible) aliphatic polyester approved by the FDA for human use that has attracted much attention in the preparation of nanoDDSs, amongst other biomedical applications (such as wound dressing, fixation devices etc.) [32,33,34,35,36,37,38]. It is a semicrystalline, hydrophobic polymer with a relatively polar ester group and five non-polar methylene groups in its repeating unit that is typically obtained by ring-opening polymerization (ROP) using caprolactone and various anionic, cationic, and co-ordination catalysts or via free radical ring-opening polymerization of 2-methylene-1-3-dioxepane [32,33]. In terms of toxicity, several animal studies have shown that PCL is a non-mutagenic and innocuous compound [39,40,41,42]. However, despite its many uses, PCL, in its linear form, shows low surface functionality and low encapsulation efficiency, while it degrades rather slowly as compared to other faster degradable polyesters (such as PLA and PLGA). As a result, in order to render PCL an ideal nanoDDS matrix/carrier, its performance must be improved by the use of appropriate structural modification.

Therefore, the aim of the present study was to evaluate, for the first time, the use of synthesized branched PCL copolymers for the preparation of drug-loaded NPs used in cancer treatment. For this purpose, three copolymers of different architectures (i.e., a three-arm PCL copolymer based on 1% *w*/*w* glycerol, PCL-GLY, a four-arm PCL copolymer based on 1% *w*/*w* pentaerythritol, PCL-PE, and a five-arm PCL copolymer based on 1% *w*/*w* xylitol, PCL-XYL) were synthesized via ring opening polymerization and fully characterized in terms of their structure, physical state, thermal properties, and cytotoxicity. Then, drug-loaded NPs were prepared by the oil-in-water emulsification/solvent evaporation method and thoroughly evaluated in regard to their physicochemical properties and dissolution characteristics. Paclitaxel (PTX), a member of the taxane family, was used as an anticancer model drug since it is considered to be one of the most useful and effective antineoplastic agents for the treatment of many forms of cancer [43].

## 2. Results and Discussion

### 2.1. Branched PCL/Polyol Copolymers Synthesis

**Scheme 1:**
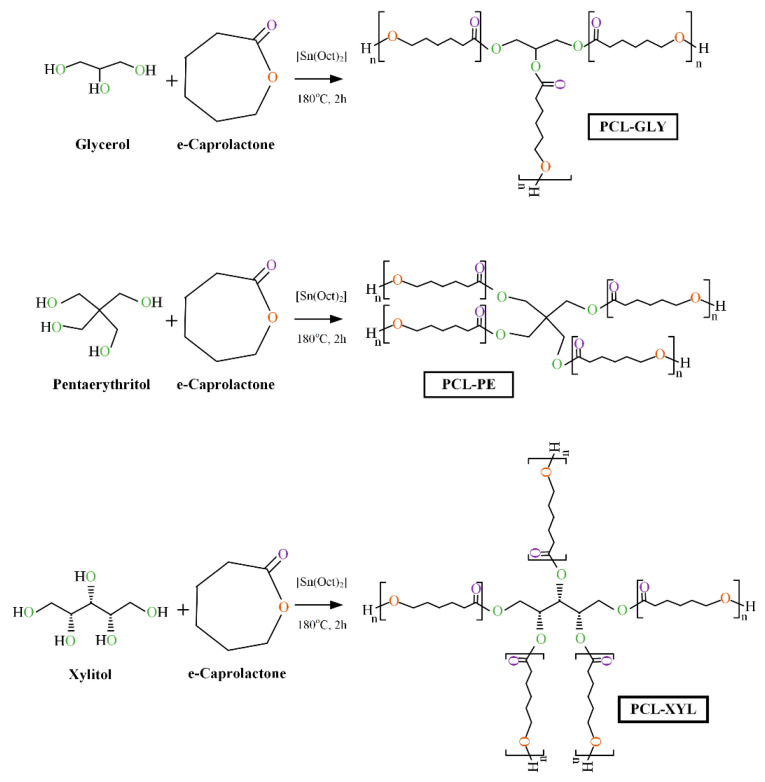
Ring-opening polymerization (ROP) reaction scheme for the synthesis of the branched PCL-based copolymers with glycerol (GLY), pentaerythritol (PE), and xylitol (XYL).

**Figure 1:**
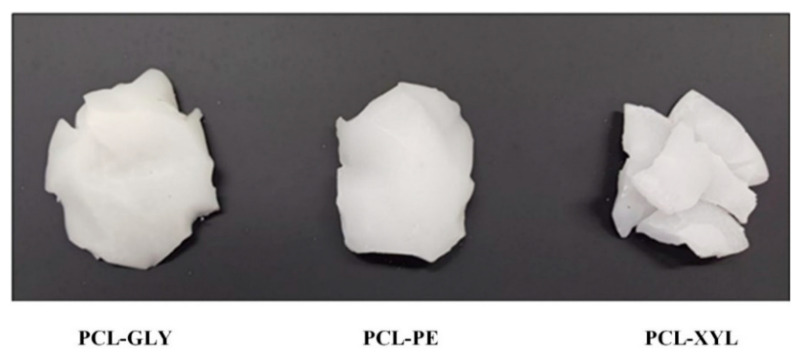
Recovered new branched PCL-based copolymers immediately after their synthesis.

In the present study, three different branched PCL-based copolymers were synthesized using three polyols (i.e., GLY, PE, and XYL). Specifically, three-arm, four-arm, and five-arm branched PCL copolyesters were synthesized via one-pot ring-opening polymerization using ε-CL as (macro)monomer and the appropriate polyol, at 1% *w*/*w*, as an initiator (Scheme 1). The reagents were dissolved in an RB flask under nitrogen, and stannous octoate was added as a catalyst. After stirring at 180 °C for 2 h, the flask was removed from the oil bath, and the reaction mixture was dissolved in dichloromethane. The contents were then precipitated into a 1/1 mixture of hexane and cold methanol and recovered by filtration. The product was dried in a vacuum at RT to constant weight, and the copolymers were obtained in all cases as wax-like solids, with white to an off-white color, as depicted in Figure 1.

#### 2.1.1. H NMR Analysis

**Figure 2:**
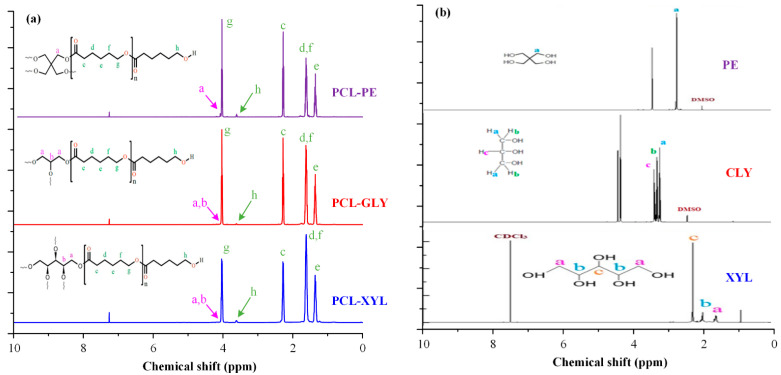
^1^H NMR spectra of three branched PCL-copolymers (**a**) and the neat polyols (**b**).

The successful synthesis of the branched PCL-copolymers was verified by ^1^H NMR (Figure 2a). In the case of the neat PCL, the ^1^H NMR spectrum of the polymer reveals several characteristic proton peaks (depicted with green) located at 4.03 ppm (corresponding to the protons (g) near the oxygen atoms), 2.27 ppm (corresponding to the protons (c) near the carbonyl group), 1.61 ppm (corresponding to the protons (d) and (f)), and 1.36 ppm (corresponding to the most shielded methylene protons (e) of the chain). These peaks were in perfect agreement with previous studies analyzing the ^1^H NMR spectra of PCL, indicating that the ROP followed in the present study led to the successful formation of the said polyester [44,45]. In the case of the synthesized copolymers, all obtained spectra showed the presence of some additional peaks (depicted with pink). Specifically, the spectra of the PCL-GLY copolymer showed two additional small peaks at 4.12 and 4.27 ppm, corresponding to the (a) and (b) protons of the GLY; PCL-PE showed a new peak at 4.08 ppm, corresponding to the (a) proton of PE; and PCL-XYL showed two new peaks at 4.07 and 4.24 ppm, corresponding the (a) and (b) protons of the XYL. In all cases, these newly recorded peaks were not seen in the initial ^1^H NMR spectra of the neat polyols (Figure 2b), indicating that the ROP reaction followed in the present study led to the formation of three new PCL-polyol-based copolymers.

#### 2.1.2. FTIR Analysis

**Figure 3:**
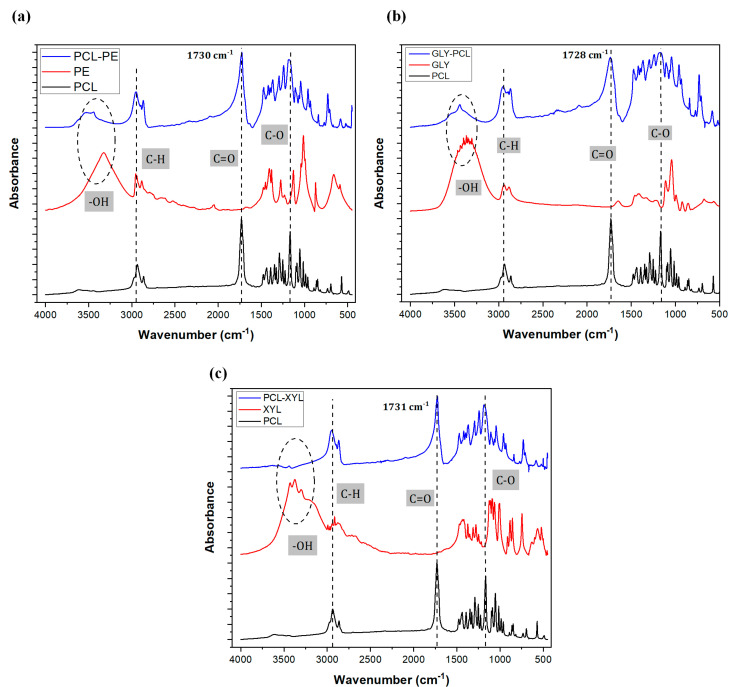
FTIR spectra of PCL and the three prepared branched PCL−copolymers: PCL−PE (**a**), PCL−GLY (**b**), and PCL−XYL (**c**).

In a further step, the successful synthesis of the PCL copolymers was also evaluated via FTIR spectroscopy (Figure 3). In the case of the neat PCL, results showed several characteristic FTIR vibrational peaks at 2950 and 2865 cm^−1^, corresponding to the C_sp3_–H asymmetric and symmetric stretching; 1731 cm^−1^, corresponding to the ester C=O stretching; and 1175 cm^−1^, corresponding to the ester C-O stretching, all of which are in perfect agreement with the FTIR spectra of PCL reported elsewhere [46,47], indicating that the polymerization reaction followed led to the successful formation of the said polyester. In the case of the synthesized branched PCL copolymers, the formation of the new ester bonds between the caprolactone monomers and the respective polyols (shown in reaction Scheme 1) is expected to show significant changes in the recorded FTIR spectra (as compared to their neat components). Specifically, in the recorded FTIR spectra of all synthesized copolyesters, a significant broadening is recorded in the peaks corresponding to the ester C=O and C-O stretching vibrations of PCL (peaks shown in 1731 and 1175 cm^−1^, respectively), while the broad peak of the neat polyols at ~3200 cm^−1^, corresponding to their hydroxyl groups, is significantly reduced in all cases. These recorded changes clearly indicate the formation of ester bonds between the -OH of the sugar alcohols and the -C=O of the e-CL monomer during the ROP reaction, thus leading to the successful synthesis of the new branched PCL copolymers.

### 2.2. Copolymer Characterization

#### 2.2.1. Molecular Weight and Intrinsic Viscosity

The average M_n_ and M_w_ of the newly synthesized PCL copolymers were determined by SEC analysis, and the results are presented in Table 1. In all cases, the M_n_ values varied between 21,116 and 43,300 g/mol, and PDI varied from 1.13 to 1.39, while unimodal peak distribution (Figure S1) was observed in all chromatograms. Additionally, low molecular weight oligomers were not detected in all three copolymers indicating that the purification steps followed after the synthetic process resulted in the formation of copolyesters with high purity.

Intrinsic viscosities results ranged from 0.294 dL/g to 0.655 dL/g, which is adequate for the preparation of DDS, as polymers (or copolymers) with higher values tend to undergo slow hydrolysis, resulting in extremely low dissolution rates for the loaded APIs.

Results show that as the number of the polyol -OH groups increase, the length of the copolymer chain is reduced. Hence, it can be suggested that the increasing number of available OH groups in the polyols results in lower intrinsic polymeric viscosity. Therefore, it seems that the presence of the hydroxyl groups in the sugar alcohols’ molecule acts as an initiator for the polymerization reaction.

#### 2.2.2. Physical State and Thermal Properties Evaluation

**Figure 4:**
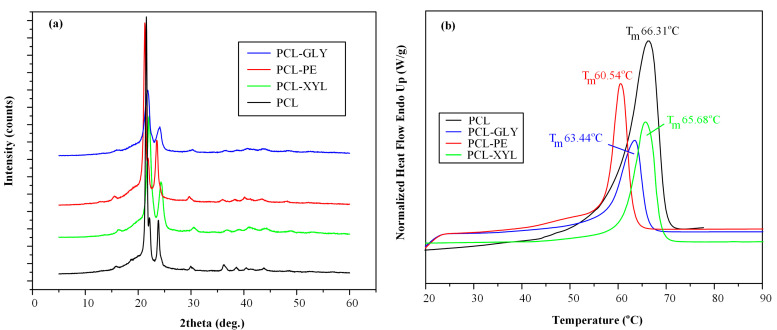
pXRD diffractograms (**a**) and DSC thermograms (**b**) and the three prepared branched PCL-copolymers.

pXRD analysis in Figure 4a showed that the synthetic process followed resulted in the formation of semicrystalline copolymers in all cases. Specifically, the neat PLC showed three characteristic pXRD peaks at 2θ of 21.45, 22.16, and 23.82°, along with a characteristic amorphous halo from ~10 to ~30°. PCL-GLY showed two characteristic pXRD peaks of lower intensity compared to the neat PCL, at 2θ of 21.78 and 24.08°; PCL-PE showed three diffractogram peaks at 21.26, 21.90, and 23.52°; slightly shifted to lower 2θ values as compared to the neat PCL; and PCL-GLY showed two peaks at 2θ of 22.08 and 24.35°, which also slightly shifted as compared to the neat PCL.

The semicrystalline character of the prepared copolyesters was also verified from the recorded DSC thermograms (Figure 4b). With respect to the neat PCL, the recorded DSC thermograms showed a single melting point at 66.31 °C, which was in agreement with previously reported results [48]. All prepared PCL copolymers showed reduced melting temperatures with Tm for PCL-XYL, PCL-GLY, and PCL-PE recorded at 65.68, 63.44, and 60.54 °C, respectively. Interestingly, based on the molecular weight results reported in Table 1, although it was expected that the depression of the melting point for the three new copolymers would occur in the following order: T_m_(PCL-GLY) > T_m_(PCL-PE) > T_m_(PCL-XYL), this was not the case.

#### 2.2.3. Enzymatic Hydrolysis

Drug release from such polyester-based DDS is mainly controlled by polymer erosion via cleavage (hydrolysis) of the ester groups. Therefore, evaluating the degree of hydrolysis is a crucial aspect of polyesters intended to be used as drug matrices. In this regard, the hydrolysis rate of the freshly synthesized copolymers was examined at 37 °C and pH 7.4 in the presence of enzymes. For that, a blend of *Rhizopus delemar* and *Pseudomonas cepacia* lipases was used since these two lipases can be triggered by adsorption on hydrophobic surfaces and therefore cleave ester bonds in the solid state.

**Figure 5:**
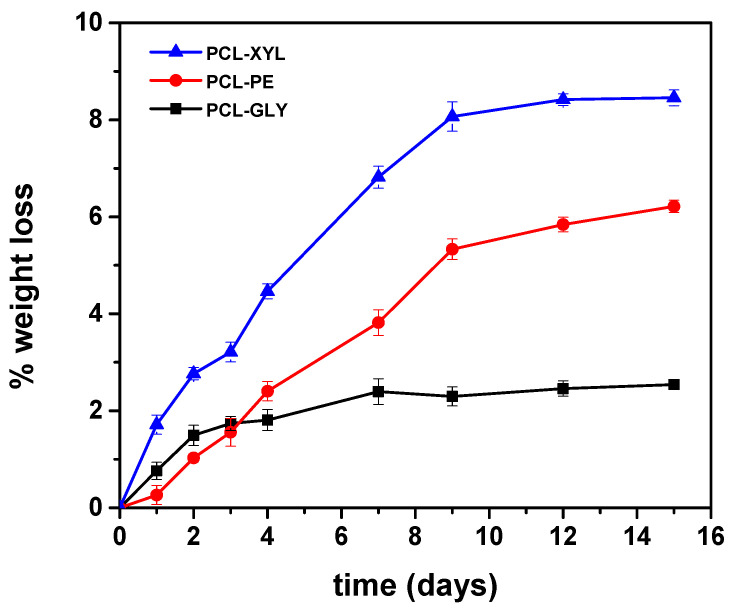
Hydrolysis rate (measured as % weight loss) of the synthesized PCL-based copolymers in the presence of *Rhizopus delemar* and *Pseudomonas cepacia* lipases.

Figure 5 demonstrates the % weight loss of the three copolymers in the presence of lipases over a period of two weeks. The results indicate hydrolysis rates varying from 2.54 to 8.45%, occurring in the following order: PCL-GLY < PCL-PE < PCL-XYL. This diverse behavior can be assigned to several parameters, such as the degree of crystallinity, the molecular weight of the polymer, the hydrophilic/hydrophobic segments ratio within the macromolecular chain, etc. In this case, the hydrolysis seems to be heavily dependent on the molecular weight and the structure of the copolymer; the lower the molecular weight and the more complex the branching, the more susceptible to hydrolysis the copolymer is. Moreover, in the case of PCL-PE and PCL-XYL, an exponential decrease in weight was observed after the third day of study that appeared to be smoothed out after a few days (~Day 9), whereas the PCL-GLY sample presented a much more stable behavior, recording a linear controlled hydrolysis profile.

**Figure 6:**
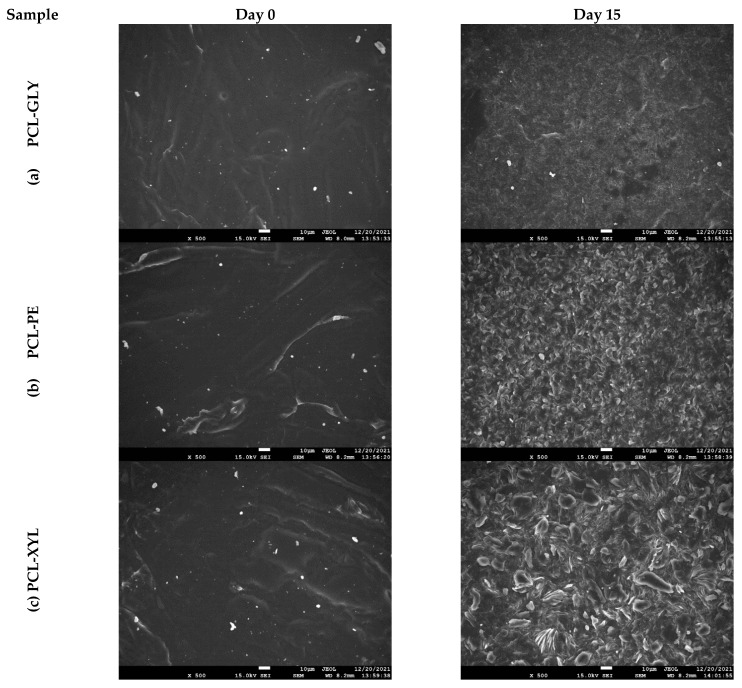
SEM images of surfaces for the PCL copolymers, initially (Day 0) and after 15 days of enzymatic hydrolysis.

Morphological evaluation of the surface of the sample using scanning electron microscopy (SEM, JEOL Ltd., Tokyo, Japan) confirmed the previously discussed results and revealed a clear image of how the degradation proceeded in each case. Figure 6 summarizes the SEM micrographs of the enzymatically hydrolyzed surfaces of the polyesters after 15 days of study. As expected, the PCL-XYL copolymer exhibited the most extensive polymer corrosion (verified by the increased surface deterioration obtained) compared to all three samples.

#### 2.2.4. Cytotoxicity Evaluation

**Figure 7:**
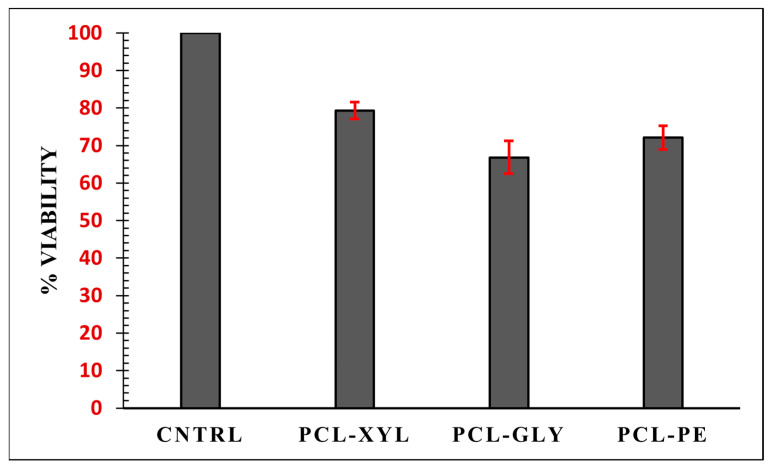
Cytotoxic effect on hAMSCs after incubation with the synthesized PCL copolymers.

When keeping in mind that all polymers (or copolymers) used as matrix/carriers in nanoDDS should possess low cytotoxicity, it is important to determine the toxicity properties of the three synthesized branched PCL-copolymer. Figure 7 shows the cytotoxicity effect of the three copolymers on the hASCs cell lines using MTT assay. During this study, the MTT passes through the mitochondria of the cells and, through this process, is reduced to formazan. The cells are then solubilized, and the amount of formazan formed during this process is quantified spectrophotometrically. As MTT reduction can only happen in metabolically active cells, the degree of activity is a measure of the cells’ viability. In general, a reduction in the measured absorbance of more than 50% relative to the control sample is required for a material to be designated as toxic. Based on this and on the obtained results, it is obvious that all studied copolyesters were non-toxic since none of them resulted in a cell viability reduction of more than 50%. Moreover, a closer look at the obtained results reveals that PCL-XYL is the copolyester with the lowest toxicity among the other two, with % a viability score of 79.33 ± 2.20%, 72.11 ± 3.13%, and 66.83 ± 4.35% for PCL-XYL, PCL-PE, and PCL-GLY, respectively.

### 2.3. PTX-Loaded NPs Characterization

According to the above analysis, the three synthesized PCL-polyol-based branched copolymers may be used as suitable matrix/carriers for the administration of anticancer APIs in the form of nanoDDS. Therefore, in the following sections, the preparation and characterization of such PTX-loaded nano-formulations were investigated in detail.

#### 2.3.1. NPs’ Morphology and Size

**Figure 8:**
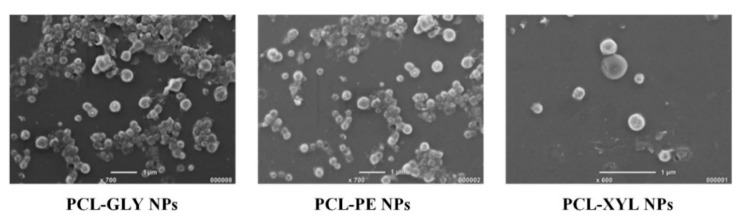
SEM images of PTX-loaded NPs prepared the synthesized PCL-branched copolymers.

Figure 8 shows the SEM micrographs of the drug-loaded NPs using the three new copolymers. Based on the obtained photographs, in all cases, the presence of well-formed particles, mostly spherical in shape, was recorded in the nanoscale (~200–300 nm), while in the cases of PCL-GLY and PCL-PE-based NPs, slightly larger size distributions were observed mostly due to the formation of agglomerates. PSD analysis via DLS showed that in all cases, NPs with sizes below 300 nm were prepared (Figure S2), with PCL-GLY-based NPs showing slightly larger hydrodynamic diameter (D_h_) values (i.e., 242 nm) as compared to PCL-PE and PCL-XYL (with D_h_ values of 224 and 236, accordingly). The recorded PDIs were within acceptable limits in all measurements with values of 0.196, 0.150, and 0.124 for PCL-GLY, PCL-PE, and PCL-XYL drug-loaded NPs, respectively.

#### 2.3.2. PTX Physical State Evaluation

**Figure 9:**
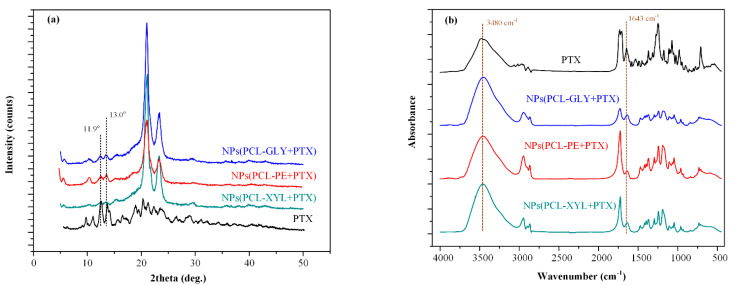
pXRD diffractograms (**a**) and FTIR spectra (**b**) of the PTX-loaded NPs prepared with the synthesized PCL branched copolymers.

pXRD (Figure 9a) was used in order to evaluate the physical state of the PCL-copolymers and the drug (PTX) after NPs preparation. In regard to the PCL-copolymers, the obtained result showed that in all cases, the copolymer characteristic pXRD peaks were recorded at the same 2θ positions as those seen in their neat form (results in Figure 4), indicating that the followed oil-in-water emulsification/solvent evaporation method did not induce any physical state changes in the selected matrix/carriers. In the case of the API, the results showed that the drug in its neat form is a highly crystalline compound with the most characteristic pXRD peaks recorded at 2θ of 11.9°, 13.0°, 18.3°, and 19.7°. As in the case of the PCL-copolymers, the pXRD diffractograms obtained in all NPs, showed all main pXRD peaks of PTX located at the same 2θ positions as in its neat form, indicating that the API also remained crystalline and unchanged (i.e., with no polymorphic transformations) during the followed encapsulation process.

#### 2.3.3. PTX-Copolymer Molecular Interactions

The formation of molecular interactions that may affect the physical state and dissolution properties of the prepared nanoDDS formulations during (or after) storage was evaluated thoroughly with the aid of FTIR spectroscopy (Figure 9b). In the case of the neat API, several characteristic FTIR peaks were recorded at 3550–3100, 2976–2888, 1732, 1643, 1245, and 1272 cm^−1^, corresponding to the -NH and -OH, -CH_2_-, -C=O, -C-O-C-, and -C-N groups of the API, respectively. With respect to the prepared NPs, all recorded spectra showed the characteristic peaks of the neat copolymers (reported in Figure 3) as well as a broad peak at 3480 cm^−1^ and a sharp peak at 1643 cm^−1^, corresponding to the -NH/-OH and the -C-O-C- groups of the API. These PTX peaks were located at the same wavenumbers as in the neat API spectra.

#### 2.3.4. Drug Loading, Encapsulation Efficiency (EE), and Yield

In order to test the efficacy of the followed NPs’ preparation method, drug loading, EE and yield were determined utilizing HPLC (method described in detail in the experimental section). All results (in terms of average values and SDs) are summarized in Table 2. Based on these, although all NPs showed a high yield value (above 85%), it seems that the type of PCL-based copolymer affected the efficacy of the process. Specifically, the use of PCL-GLY resulted in a 5% and 10% increase in NPs’ yield as compared to the PCL-XYL and PCL-PE, respectively. Interestingly, this order was reverted in terms of EE, with PCL-XYL showing 6% and 12% higher EEs as compared to PCL-PE and PCL-GLY.

These results indicated that as the branched network of the prepared PCL-polyol copolymer becomes more complex, the drug is more effectively trapped within its structure. In any case, the high EE values obtained in all cases, along with the high yield values, indicate that the prepared branched copolymers were able to overcome the low encapsulation efficiency limitation that is associated with the use of linear PCL (or any other type of linear polyester).

#### 2.3.5. In Vitro Drug Dissolution Studies

**Figure 10:**
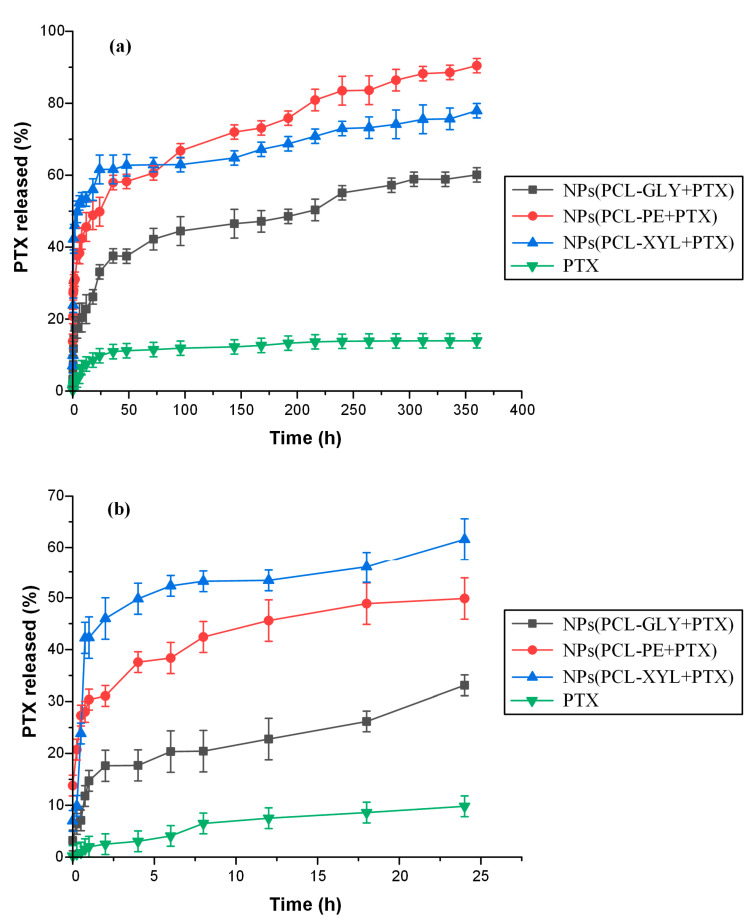
In vitro dissolution profiles of the neat PTX and the PTX-loaded NPs prepared with the synthesized PCL branched copolymers up to 15 days (**a**) and 1 day (**b**), respectively.

Figure 10a shows the in vitro dissolution profiles for the neat PTΧ, and the PΤΧ-loaded NPs prepared with the synthesized PCL branched copolymers (i.e., PCL-GLY. PCL-PE and PCL-XYL) in phosphate buffer saline (PBS) medium (pH = 7.4) as monitored via HPLC over a period of 15 days (360 h). Based on the obtained results, the pure drug showed a limited dissolution extent, reaching a maximum of about ~15% within the first two days and no further increase thereafter, a result that can be attributed to its low aqueous solubility [49,50,51]. On the contrary, looking at the obtained dissolution profiles for the NPs, all formulations achieved a significant improvement in API’s dissolution characteristics, with maximum dissolved API reaching in these cases ~55%, ~75%, and ~90% for the NPs prepared with PCL-GLY, PCL-XYL, and PCL-PE, respectively. Hence, from the obtained results, it can be said that the formation of PTX-loaded NPs using the proposed branched PCL copolymers resulted in PTX’s significant solubility and in vitro dissolution enhancement.

When looking more closely at the obtained results, it is obvious that all NP formulations followed a biphasic release profile, with an initial fast release (burst effect) seen in the first 4–5 h, followed by a sustained release until the end (i.e., 15 days). The burst release observed initially can be attributed to the drug placed/absorbed on the surface of the NPs, while the following slow-release rate is probably due to the polyester’s glass–rubbery transition leading to variations in two moving release controlling fronts, i.e., swelling and erosion/degradation [52]. Nevertheless, results regarding the initial fast release presented in Figure 10b showed that PTX’s burst effect was dependent on the type of polyester used, with PCL-XYL-based NPs showing the highest burst release, ~50% of the API was released in the first 5 h, followed by the PCL-PE and PCL-GLY with ~40% and ~20% of the API being released in the same timeframe. Thus, it seems that as the branched network of the PCL-polyol copolymer is becoming more complicated, the API struggles to penetrate into the NPs’ structure leading to a higher percentage of drug located on the surface of the particles, leading thus to higher initial release rates and burst release. After this first stage of the immediate (burst) release, all NPs showed a zero-order release profile starting from approximately 24 h until the end of the dissolution test (R^2^ on the fitting of the zero-order release kinetics model [53] was above 0.97 in all cases). Interestingly, the NPs prepared with PCL-XYL showed a much lower release rate as compared to the other two polyesters, indicating that in this case (i.e., the sustained release phase), the more complex structure of the copolymer probably leads to significant steric hindrance phenomena that restrict drug’s movement within the swollen NPs’ structure and hence, restrict its interactions with the molecules of the dissolution medium leading to its solubilization.

#### 2.3.6. Modeling of Release Kinetics

Towards a further discussion on the previously collected data, an attempt to quantify the observed release kinetics following the above mechanisms was made here. Typically, a dissolution curve is modeled by using the Noyes–Whitney [54] equation or its extension [55]. Under the selected dissolution conditions, it is expected that the dominant drug release step is the phase transition of the drug from the solid to its dissolved state. However, in the case of the neat API dissolution, when the dominant linear equation is solved, the fit is not adequate, so an extension of the simple exponential expression is needed. For this reason, a two-exponential expression of the following form was considered:

(1) $$R_{c}=R_{o}\left(1-\varphi_{1}e^{-k_{1}t}-\left(1-\varphi_{1}\right)e^{-k_{2}t}\right)$$

where *R* is the % release of the drug up to time t, *R_o_* is the final drug release (determined by the drug solubility), *φ*_1_ is the fraction of drug dissolved, and *k*_1_ and *k*_2_ are the rate constants.

**Figure 11:**
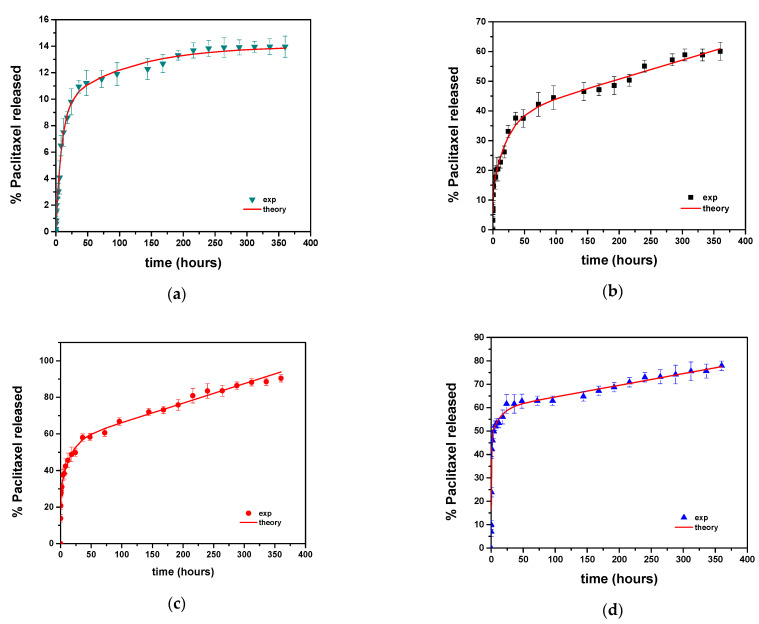
Comparison between experimental release data (symbols) and fitted curves (lines): (**a**) pure drug; (**b**) PCL-GLY; (**c**) PCL-PE; (**d**) PCL-XYL nanoparticles.

The fitting of the experimental dissolution data for the neat API using this two-exponential equation was excellent (*R*^2^ > 0.99, *R_o_* = 14%, *φ*_1_ = 0.33, *k*_1_ = 9.4 × 10^−3^ h^−1^ and *k*_2_ = 0.113 h^−1^). The comparison between experimental data and the fitting of the API dissolution curve is shown in Figure 11a.

When looking at the drug’s biphasic release profiles from the polymeric matrices, it is obvious that the shape of the release data is too complex to fit into the general functional forms used extensively in the literature [56]. As previously discussed, the release appears to be determined by a combination of diffusion, erosion, and drug distribution within or on the surface of the polymeric matrix. The first issue to be examined is whether the experimentally observed initial burst release of the API from the polymeric particles (i.e., the first phase of dissolution) can be attributed to the well-known ‘diffusion burst’ or it is the non-uniform drug distribution (i.e., excess amounts of drug located near the surface to the particles) that explains this behavior. For this purpose, the diffusion equation for the spherical geometry (assuming uniform spatial drug distribution) was considered [57]:

(2) $$R_{c}=R_{o}\left(1-\frac{6}{\pi^{2}}\overset{\infty }{\underset{i=1}{\sum }}\frac{1}{i^{2}}\exp(-i^{2}\pi^{2}Kt\right))$$

where *K* is a rate constant depending on (1) the drug diffusivity into the polymer and (2) the particle size.

Fitting of the API burst release data on Equation (2) was not successful, indicating that the initial burst drug release observed from all polymeric matrices was due to the excess amount of the drug located near the surface of the polymeric particles. Therefore, it can be assumed that this excess drug amount was initially released via a fast diffusion mechanism due to the close proximity of the drug particles to the liquid (dissolution) surface.

When moving to the second dissolution phase of the biphasic drug release profile, it seems that the drug (which is now uniformly distributed within the polymeric matrix) is released through a combination of Fickian diffusion and polymer matrix erosion. A close observation of the experimental data suggests that the erosion and diffusion mechanisms occur in different timescales; thus, it can be assumed that these two mechanisms are independent of each other. In this case (i.e., independent diffusion and erosion), the diffusion equation derives from the linear driving force formula (LDF), which leads to single exponential dependencies [58].

By summarizing the above considerations, the equation involving both dissolution phases (i.e., the initial burst and the diffusion/erosion-driven drug release) is given below:

(3) $$R_{c}=\varphi_{1}\left(1-\exp(-K_{1}t\right))+\varphi_{2}\left(1-\exp(-K_{2}t\right))+K_{3}t$$

where *φ*_1_ and *φ*_2_ are the percentage fractions of the drug in the surface excess layer and in the uniformly distributed region, respectively. The corresponding release rate constants are *K*_1_ and *K*_2_, respectively. Finally, the erosion rate constant is denoted as *K*_3_.

The fitting of the experimental data on Equation (3) shows high *R*^2^ values (>0.98), indicating that the assumptions made for the preparation of the mathematical model were adequate. The comparison between experimental data and fitting curves in all cases is shown in Figure 11b–d. The optimum parameter values resulting from the fitting procedure are summarized in Table 3.

Based on the above table, it is noted that, in most cases, the drug content on the surface layer, *φ_1_*, exceeds the uniformly distributed drug fraction corresponding to *φ_2_*. In addition, the erosion rate was higher for the PCL-PE matrix and smaller (comparable to each other) for the other two materials. Finally, an estimation of the diffusion coefficient, D, in each case, can be made from the following equation: *K* = 15 D/r^2^ (deriving from LDF approximation [58]), where r is the particle radius. Based on this, the D values were estimated as 2 × 10^−20^ m^2^/s, 3.31 × 10^−20^ m^2^/s, and 1.56 × 10^−20^ m^2^/s for the PCL-GLY, PCL-PE, and PCL-XYL matrices, respectively, which were in the typical scale derived for such drug/polymer diffusion/erosion-driven release matrices.

## 3. Materials and Methods

### 3.1. Materials

PTX, a white, odorless, crystalline powder with 99.5% purity, and MW of 853.91 g/mol, was purchased from Alfa Aesar (CAS Number: 33069-62-4) and was donated by Pharmathen S.A. (Athens, Greece). The monomer ε-caprolactone (ε-CL, purity 99%), Tin(II) 2-ethylhexanoate (TEH) catalyst (analytical grade), polyvinyl alcohol (PVA), poly(ethylene glycol), pentaerythritol (reagent grade 99%), glycerol (ACS reagent Z99.5%), and xylitol (≥99%) were purchased from Sigma-Aldrich (Saint Louis, MO, USA). All the other reagents used were of either analytical or pharmaceutical grade and used as received.

### 3.2. Preparation of the Synthesized Branched PCL Copolymers

Initially, the neat PCL polymer was synthesized via ROP using the ε-CL monomers based on a previously published method [59]. The monomer (ε-CL) was placed in a round-bottom flask (250 mL), and the polymerization process was conducted there under a high vacuum. TEH, solubilized in toluene at a 1 × 10^−4^ mole per mole concentration with respect to the e-CL monomer, was used as a catalyst, while nitrogen (N_2_) was used for purging the solution three times. The components were left to react at 190 °C for three (3) hours, and then the temperature was increased to 240 °C over a period of 90 min. Polymerization was terminated by rapid cooling to room temperature. After completion of the polymerization reaction, the non-reacted monomers were removed via distillation.

The new branched PCL copolymers were also synthesized via ROP following a previously published method [59]. Briefly, in the case of the PCL-CLY copolymer, the ε-CL monomer, along with appropriate amounts of GLY (at a 1% *w*/*w* concentration with respect to the e-CL monomer) was placed in suitable round bottom flask reactors and heated at 150 °C under inert atmosphere (N_2_) in order to ensure the complete solubilization of GLY in the respective monomer. Then, THE was added, and the polymerization reaction proceeded at 160 °C for 6 h under a high vacuum. A similar procedure was also followed for the synthesis of the PCL-PE and PCL-XYL, with the amount of pentaerythritol and xylitol added to the reaction being fixed to 1% *w*/*w* of e-CL in both cases.

### 3.3. Characterization of the Synthesized Branched PCL Copolymers

#### 3.3.1. Nuclear Magnetic Resonance Spectroscopy

The ^1^H NMR spectra of all prepared materials were collected using an Agilent spectrometer (Santa Clara, CA, USA) operating at 500 MHz for protons at room temperature. Deuterated chloroform (CDCl_3_) was used as a solvent to prepare solutions of 5% *w*/*v*. The number of scans was 16, and the sweep width was 6 kHz.

#### 3.3.2. Intrinsic Viscosity

Intrinsic viscosity, *η*, measurements were performed by using an Ubbelohde 0c viscometer (witeg Labortechnik GmbH, Wertheim, Germany) at 25 °C. Briefly, all samples were dissolved in chloroform (1% *w*/*v*) at room temperature and filtered through a 0.2 mm, Teflon filter, and the intrinsic viscosity was calculated using Equation (4) [60]:

[η] = [2(t/t0 − ln(t/t0) − 1)]1/2/c (4)

where c is the concentration of the solution, t is the flow time of the solution, and to is the flow time of pure solvent.

#### 3.3.3. Powder X-Ray (pXRD) Diffractometry

The pXRD spectra of PCL and all prepared copolymers were recorded by means of a MiniFlex II XRD system (Rigaku Co., Tokyo, Japan), with Cu Ka radiation (l = 0.154 nm), over the 2θ range from 5° to 60° with a scanning rate of 1 deg min^−1^.

#### 3.3.4. Fourier-Transform Infrared (FTIR) Spectroscopy

FTIR spectra were recorded using a PerkinElmer FT-IR spectrometer (Spectrum 1, Waltman, MA, USA) on KBr containing tablets of the samples. The spectra were obtained over the range of 4000 to 400 cm^−1^, at a resolution of 4 cm^−1^, using 16 co-added scans. All spectra presented are baseline-corrected, normalized, and converted to absorbance mode.

#### 3.3.5. Differential Scanning Calorimetry (DSC)

A Pyris Diamond DSC apparatus (Perkin–Elmer, Waltham, MA, USA) was used in order to record the thermal properties of the synthesized polyesters. The instrument was calibrated with sapphires for heat capacity and indium for temperature and enthalpy. During the measurement, the samples were initially heated to 100 °C and stayed there for ~3 min in order to erase any thermal history and to evaporate any remaining humidity or solvents (scan 1). Then, the melted samples were cooled at −150 °C/min (with a cooling rate of 10 °C/min) and heated up again to 100 °C at the same rate (scan 2).

#### 3.3.6. Size Exclusion Chromatography (SEC)

Molecular weight determinations were performed using a high-temperature SEC system equipped with a Waters pump (model 600, Milford, MA, USA), a Shimadzu refractive index detector (RID 10-a, Kyoto, Japan), and Water columns (Styragels, Milford, MA, USA) in the order of HR1, HR2, HR4, HR4, and HR5. All samples were dissolved in CHCl_3_ at a constant concentration of 12 mg/600 μL and filtrated. The elution solvent was CHCl_3_ at a constant flow rate of 1 mL/min, and the measurements were performed at 35 °C and 200 μL injection volume. Calibration was performed using nine polystyrene standards with narrow molecular weight distribution, 1000–300,000 molecular weight distribution.

#### 3.3.7. Enzymatic Hydrolysis

Polymer biodegradation describes the chain scission process during which polymer chains are cleaved in the form of oligomers and monomers, resulting in polymer erosion and mass loss. In the present study, for the evaluation of the degradation process, the produced polyesters in the form of films (5 × 5 cm and ~2 mm thickness) were prepared by a PW 30 Otto Weber (Dusseldorf, Germany) hydraulic press connected to a temperature controller (Omron E5AX, Dusseldorf, Germany). The samples were incubated at 37 ± 1 °C for 15 days in suitable Petri dishes containing phosphate-buffered saline (PBS) (pH = 7.4) with Rhizopus oryzae and Pseudomonas cepacia lipases at 0.09 and 0.01 mg/mL content, respectively. After specific period intervals, the films were removed from the Petri dishes, washed with distilled water, and weighted until a constant weight was achieved. The degree of biodegradation was estimated from the mass loss of the weighted polymer.

Surface morphology characterization of the prepared films before and after the enzymatic hydrolysis was evaluated using field-emission scanning electron microscopy, JEOL JSM-7610F Plus (JEOL Ltd., Tokyo, Japan), supported by an Oxford AZTEC ENERGY ADVANCED X-act energy dispersive X-ray spectroscopy (EDS) system (JEOL Ltd., Tokyo, Japan). A thick carbon coating was applied to the obtained films to increase the conductivity of the samples. The accelerating voltage, the probe current, and the counting time were set at 20 kV, 45 nA, and 60 s, respectively.

#### 3.3.8. Cytotoxicity Studies

The MTT cell proliferation assay, which employs the reduction in tetrazolium salts by metabolically active cells for examining cellular viability, was used for the in vitro cytotoxicity assessment of the prepared copolymers (Trevigen, Gaithersburg, MD, USA 4890-025-K). Human adipose-derived mesenchymal stem cells (hAMSCs) provided by Biohellenika S.A. (Thessaloniki, Greece) after adipose tissue isolation from healthy volunteer donors were used for the evaluation. All materials were sterilized in gradually reduced ethanol concentrations (100%, 70%, and 50% in ddH_2_O) and, after washing twice with ddH_2_O, were left to air dry for 5 h under sterile conditions. Filters of 0.22 μm pore size were used for the sterilization. Accurately weighted samples of 200 μg/mL concentration were then prepared in Dulbecco’s modified Eagle’s medium (DMEM) (BIOWEST, Nuaillé, France) supplemented with 10% FBS and 2% penicillin/streptomycin to be added directly onto the cell culture. After 48 h of coincubation with the formulations, the medium was removed, and cells were washed once with PBS before adding the fresh medium, including the 1/10 MTT reagent (Sigma-Aldrich, Saint Louis, MI, USA). Upon the removal of the MTT, 1 mL/well of DMSO was introduced for one additional hour of incubation. The optical density of MTT formazan deposits was quantified by a spectrophotometer at 570 nm and 630 nm (UV-vis spectrophotometer: Perkin Elmer, Dresden, Germany), while viability percentage was calculated as {[Sample’s OD_570/630_] × 100}/Control OD_570/630_. All experiments were conducted in triplicate.

### 3.4. Preparation of NPs

PTΧ-loaded polymeric nanoparticles were prepared by the oil-in-water emulsification/solvent evaporation method. Briefly, 200 mg of the polyesters and 20 mg of PTΧ were dissolved in 5 mL of dichloromethane (DCM) under sonication for 1 min. Then the resultant solution (oil phase) was homogenized with an aqueous phase (40 mL) consisting of 0.5% *w*/*v* PVA using probe sonication UP100H (Hielscher Ultrasonics, Oderstraße, Teltow, Germany) for 2 min and transferred to 100 mL of deionized water, where it was left for 24 h at 25 °C under mild agitation. The resultant nanoparticles were collected after centrifuging at 12,000 rpm for 10 min and washed three times with ultrapure water in order to remove the remaining quantities of DCM and PVA. Finally, the microspheres were freeze-dried in order to remove the excess water and stored at 4 °C before further analysis. Unloaded NPs (i.e., NPs without the API) were prepared following the same preparation method.

### 3.5. Characterization of NPs

#### 3.5.1. Physical State Evaluation

The physical state of the copolymers and the API was determined via pXRD analysis using the same method and organology described in Section 3.3.3.

#### 3.5.2. Evaluation of Molecular Interactions

The formation of molecular interactions between the API and the used branched copolymers during the preparation of NPs was evaluated via FTIR spectroscopy using the same method and organology described in Section 3.3.4.

#### 3.5.3. Evaluation of Particle Size Distribution (PSD) and NPs’ Morphology

NPs’ PSD (and PDI) was determined by dynamic light scattering (DLS) using a Zetasizer Nano-S system by Malvern Instruments (Malvern, UK). For the preparation of samples, a 100-fold dilution with low ionic strength (2 mM) phosphate buffer at pH 7 was performed. All measurements were conducted at 25 °C in triplicate.

NP’s morphology was evaluated via scanning electron microscopy (SEM). Briefly, SEM images were acquired with an ultra-high resolution Schottky Field Emission Scanning Electron Microscope, model JSM-7610, by JEOL (Akishima, Tokyo, Japan). For the measurements, a drop of each NPs (suspended in water) was placed in the holder and left to evaporate. All samples were covered with carbon to establish good conductivity. During the measurements, the SEMs operating conditions were: 20 kV accelerating voltage, 45 nA probe current, and 60 s counting time.

#### 3.5.4. Drug Loading, Encapsulation Efficiency (EE), and Yield

PTX’s drug loading and EE were measured by dissolving 10 mg of NPs in 10 mL of dichloromethane:methanol (1:1 *v*/*v*) and determining the drug’s assay via HPLC (method details are given below). Drug loading, yield, and entrapment efficiency were calculated by using the following equations:

(5) $$\mathrm{Drug}\;\mathrm{loading}\;\left(\%\right)=\frac{\mathrm{weight}\;\mathrm{of}\;\mathrm{PTX}\;\mathrm{in}\;\mathrm{NPs}}{\mathrm{weight}\;\mathrm{of}\;\mathrm{NPs}}\times 100$$

(6) $$\mathrm{Yield}\;\left(\%\right)=\frac{\mathrm{weight}\;\mathrm{of}\;\mathrm{NPs}}{\mathrm{initial}\;\mathrm{weight}\;\mathrm{of}\;\mathrm{raw}\;\mathrm{materials}}\times 100$$

(7) $$\mathrm{EE}\;\left(\%\right)=\frac{\mathrm{weight}\;\mathrm{of}\;\mathrm{PTX}\;\mathrm{in}\;\mathrm{NPs}}{\mathrm{initial}\;\mathrm{weight}\;\mathrm{of}\;\mathrm{PTX}}\times 100$$

#### 3.5.5. In Vitro Dissolution Studies

The in vitro release studies were performed in a DISTEK Dissolution Apparatus I (Dissolution system 2100C, Distek, North Brunswick, NJ, USA) equipped with an autosampler (Evolution 4300, Distek, North Brunswick, NJ, USA). Appropriate dialysis tubing cellulose membranes were used in all cases. All tests were executed at 37 ± 1 °C with 50 rpm. The dissolution medium was 500 mL of simulated body fluid (SBF) at pH = 7.4. Two milliliters of aqueous solution were withdrawn from the release media at predefined time intervals (i.e., 15, 30, 45 min and 1, 2, 4, 6, 8, 12, 18, 24, 36, 48, 72, 96, 120, 144, 168, 192, 216, 240, 264, and 288 h) and the API was quantified via the HPLC method described below.

#### 3.5.6. HPLC Analysis

PTX content was determined using a Shimadzu HPLC prominence system (Kyoto, Japan) consisting of a degasser (DGU-20A5, Kyoto, Japan), a liquid chromatograph (LC-20 AD, Kyoto, Japan), an autosampler (SIL-20AC, Kyoto, Japan), a UV/Vis detector (SPD-20A, Kyoto, Japan), and a column oven (CTO-20AC, Kyoto, Japan). An Eclipse XDB-C18 5 μm, 250 mm × 4.6 mm analytical column was used, while the flow rate was set at 1 mL/min, and the column temperature was maintained at 25 °C. A diode array detector was used at 227 nm, and the quantification of the API was based on a calibration curve prepared at 20, 10, 5, 2.5, 1, and 0.5 μg/mL API to mobile phase (water/ACN 30/70 *v*/*v*).

## 4. Conclusions

In the present study, new branched PCL-polyol copolymers were synthesized via ROP and used for the preparation of PTX-loaded NPs. All synthesized copolyesters were semicrystalline in nature and non-toxic, while the presence of the hydroxyl groups in the polyols’ molecule acted as a macroinitiator for the polymerization reaction. The prepared drug-loaded NPs were mostly spherical, with sizes ranging from 200 to 300 nm, while high yield and EE results (>60%) were obtained in all cases. In addition, API crystals were dispersed within all tested copolymeric matrices, while the use of the new branched PCL copolymers resulted in PTX’s significant in vitro dissolution enhancement as compared to the pure drug alone. In any case, results in the present study showed that the prepared branched PCL-polyol copolymers were able to overcome the limitations traditionally encountered by the use of linear polyesters, and hence, they can be considered a promising alternative for the preparation of a successful and efficient anticancer nanoDDS.

## Figures and Tables

**Table 1:** Molecular weights, PDI, and intrinsic viscosities of the synthesized PCL-copolymers.

Sample	M_n_ (g/mol)	M_w_ (g/mol)	PDI
PCL-GLY	43,300	60,400	1.39
PCL-PE	29,000	32,900	1.13
PCL-XYL	21,116	26,438	1.25

**Table 2:** NPs’ yield, drug loading, and encapsulation efficiency (EE).

Sample	Yield (%)	Drug Loading (%)	EE (%)
NPs(PCL-GLY + PTΧ)	96 ± 1.38	5.97 ± 1.39	60.09 ± 2.02
NPs(PCL-PE + PTΧ)	90 ± 2.56	6.58 ± 2.02	66.26 ± 2.85
NPs(PCL-XYL + PTΧ)	86 ± 1.84	7.42 ± 1.98	72.01 ± 2.70

**Table 3:** Equation (3) fitting parameters for the drug-polymer dissolution profiles.

Sample	*φ*_1_	*φ*_2_	*K*_1_ (h^−1^)	*K*_2_ (h^−1^)	*K*_3_ (h^−1^)
PCL-GLY	14.1	23.9	1.95	0.043	0.064
PCL-PE	29	26.4	4.65	0.066	0.106
PCL-XYL	47.7	11.9	1.68	0.072	0.05

## Supplementary Materials

The following supporting information can be downloaded at: https://www.mdpi.com/article/10.3390/ijms232315393/s1.
