# Supplementary material for: Branched Poly(ε-caprolactone)-Based Copolyesters of Different Architectures and Their Use in the Preparation of Anticancer Drug-Loaded Nanoparticles
Source: Int J Mol Sci. 2022 Dec 6;23(23):15393. doi: 10.3390/ijms232315393 (PMC9735713; doi:10.3390/ijms232315393)
Supplement: Supplementary file 1 [file ijms-23-15393-s001.zip › ijms-2051294-supplementary.pdf]

International Journal of
Molecular Sciences

MDPI

Article

Branched Poly(*E*-Caprolactone)-Based Copolyesters of Different Architectures And Their Use IN the Preparation of Anticancer Drug-Loaded Nanoparticles

Evi Christodoulou [1], Maria Notopoulou [1], Eirini Nakiou [1], Margaritis Kostoglou [2], Panagiotis Barmpalexis [3] and Dimitrios N. Bikiaris [1,*]

[1] Laboratory of Polymer Chemistry and Technology, Department of Chemistry, Aristotle University of Thessaloniki, Thessaloniki 54124, Greece; evicius@gmail.com (E.C.); marynoto@hotmail.com (M.N.); renia.nakiou@hotmail.com (R.N.); dbic@chem.auth.gr (D.N.B.)
[2] Laboratory of General and Inorganic Chemical Technology, Department of Chemistry, Aristotle University of Thessaloniki, Thessaloniki 54124, Greece; kostoglu@chem.auth.gr (M.K.)
[3] Department of Pharmaceutical Technology, School of Pharmacy, Aristotle University of Thessaloniki, Thessaloniki 54124, Greece; pbarmp@pharm.auth.gr (P.B.)

* Correspondence: dbic@chem.auth.gr; Tel. +30 2310 997812 (D.N.B.)

SUPPLEMENTARY INFORMATION

1. *Molecular weight estimation*

The molecular weights of the copolymers were estimated by Gel Permeation Chromatography/Size Exclusion Chromatography (GPC/SEC). The analysis was performed by means of a GPC/SEC equipment composed of a Waters 600 high pressure liquid chromatographic pump, Waters Ultrastyragel columns (HR-1, HR-2, HR-4, HR-5), and a Shimadzu RID-10A refractive index detector. Column calibration was performed using polystyrene standards (Mw 1–300k). The concentration of the prepared solutions was 10 mg/700 μL, the injection volume was 200 μL and the flow rate 1 mL/min.

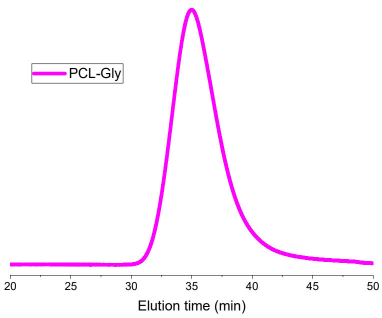

(a)

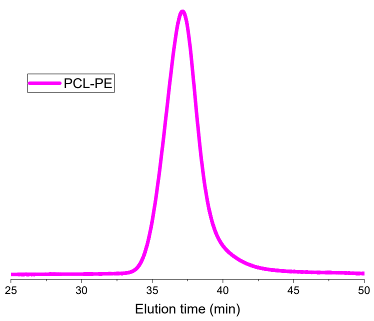

(b)

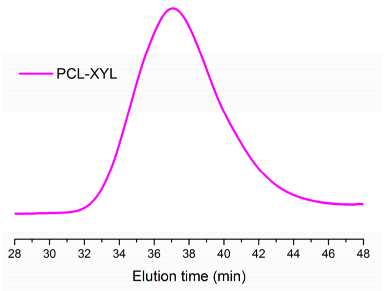

(c)

Figure S1. Size-exclusion (SEC) chromatograms of the prepared copolyesters.

2. Nanoparticles size determination

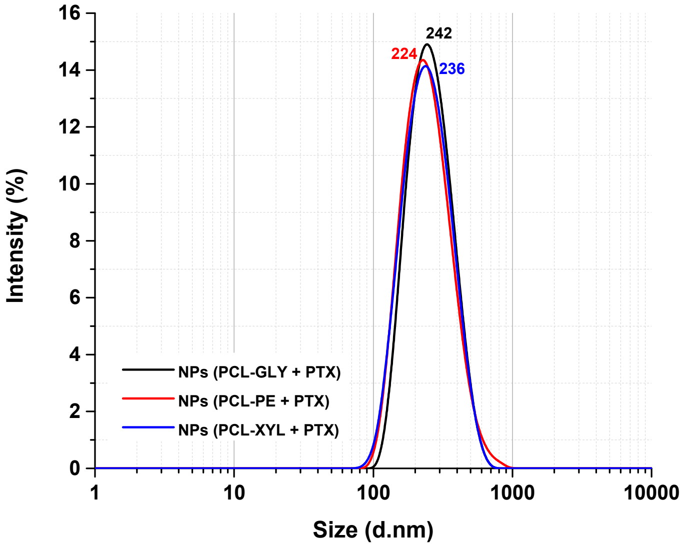

Figure S2. Particle size measurements by dynamic light scattering measurements.
